# Endothelial cilia dysfunction in pathogenesis of hereditary hemorrhagic telangiectasia

**DOI:** 10.3389/fcell.2022.1037453

**Published:** 2022-11-10

**Authors:** Shahram Eisa-Beygi, Patricia E. Burrows, Brian A. Link

**Affiliations:** ^1^ Department of Cell Biology, Neurobiology, and Anatomy, Medical College of Wisconsin, Milwaukee, WI, United States; ^2^ Department of Radiology, Medical College of Wisconsin, Milwaukee, WI, United States

**Keywords:** Vascular disease, Zebrafish, TGF-β, Endothelial cilia, BMP signaling

## Abstract

Hereditary hemorrhagic telangiectasia (HHT) is associated with defective capillary network, leading to dilated superficial vessels and arteriovenous malformations (AVMs) in which arteries connect directly to the veins. Loss or haploinsufficiency of components of TGF-β signaling, ALK1, ENG, SMAD4, and BMP9, have been implicated in the pathogenesis AVMs. Emerging evidence suggests that the inability of endothelial cells to detect, transduce and respond to blood flow, during early development, is an underpinning of AVM pathogenesis. Therefore, components of endothelial flow detection may be instrumental in potentiating TGF-β signaling in perfused blood vessels. Here, we argue that endothelial cilium, a microtubule-based and flow-sensitive organelle, serves as a signaling hub by coupling early flow detection with potentiation of the canonical TGF-β signaling in nascent endothelial cells. Emerging evidence from animal models suggest a role for primary cilia in mediating vascular development. We reason, on recent observations, that endothelial cilia are crucial for vascular development and that embryonic loss of endothelial cilia will curtail TGF-β signaling, leading to associated defects in arteriovenous development and impaired vascular stability. Loss or dysfunction of endothelial primary cilia may be implicated in the genesis of AVMs due, in part, to inhibition of ALK1/SMAD4 signaling. We speculate that AVMs constitute part of the increasing spectrum of ciliopathy-associated vascular defects.

## Introduction

Hereditary hemorrhagic telangiectasia (HHT) is characterized by vascular lesions that lack a normal capillary network (Braverman et al., 1990; Mahmoud et al., 2010; Peacock et al., 2016), leading to development of dilated superficial vessels (telangiectasias) and arteriovenous malformations (AVMs), (Jessurun et al., 1993; Ference et al., 1994; McDonald et al., 2000; Shovlin et al., 2000; Willemse et al., 2000), particularly, in vessels experiencing high fluid shear stress (Corti et al., 2011; Baeyens et al., 2016; Ola et al., 2018). AVMs are focal or diffuse abnormalities in which arteries connect directly to veins, manifesting in venous hypertension with engorgement. Involving mainly the central nervous system (CNS) and viscera in this condition, they lead to right-to-left shunting in the lungs and carry a high risk of hemorrhages. Furthermore, impaired perivascular coverage is a hallmark of hemorrhage-prone lesions in both AVMs and HHT (Li et al., 2011; Chen et al., 2013; Thalgott et al., 2015; Baeyens et al., 2016; Winkler et al., 2018; Zhu et al., 2018). In HHT patients, hemorrhages are most commonly detected in telangiectasias in mucous membranes and rarely in the lung or brain. The etiology and pathogenesis of AVMs are thought to involve, at least in part, loss-of-function and/or haplo-insufficiency of the well-conserved components of the transforming growth factor beta (TGF-β) pathway, resulting in curtailment of BMP9-mediated signaling (Ricard et al., 2010; Cunha et al., 2017). In particular, loss-of-function mutations in the membrane proteins, activin A receptor-like kinase 1 (ALK1/ACVRL1), Endoglin (ENG) (Johnson et al., 1996; Berg et al., 1997; Shovlin et al., 1997; Pece et al., 1997; Letteboer et al., 2005; Schulte et al., 2005; Lesca et al., 2006; Fernandez-L et al., 2006; Lenato et al., 2006; Sabbà et al., 2007; Albiñana et al., 2017; Ruiz-Llorente et al., 2017; Mallet et al., 2015; Hume et al., 2013; Plumitallo et al., 2018), and, to a lesser frequency, mutations in SMAD4 and BMP9, have been implicated in some forms of HHT and HHT-like vascular manifestations that present with variable penetrance and heterogeneous phenotypic manifestations (Gallione et al., 2006; Gallione et al., 2010; Iyer et al., 2010; Wooderchak-Donahue et al., 2013; Tillet and Bailly, 2015; Jelsig et al., 2016; Vorselaars et al., 2017; Karlsson and Cherif, 2018; Balachandar et al., 2022).

Evidence from animal studies suggest that upon activation by circulating growth factors, BMP9 and BMP10, ALK1, through its interaction with its co-receptor ENG, phosphorylates the downstream effectors, SMAD1/5/8, resulting in translocation of the downstream SMAD4 to the nucleus. In brief, both ligands bind to ALK1 and the two type II TGFβ receptors where ENG serves as a co-receptor. Upon dissociation of ENG from the ligand, ALK1 is phosphorylated, and this is followed by phosphorylation of SMAD1, SMAD5, or SMAD9 phosphorylation. In the nucleus, the p-SMAD1/5/8 and SMAD4 complex functions as a transcription factor to regulate the expression of target genes. The ALK1/SMAD4 signaling has been implicated in endothelial cell (EC) quiescence, vascular stabilization, and recruitment of perivascular cells (David et al., 2008; Baeyens et al., 2016; Franco and Gerhardt, 2016; Akla et al., 2018). However, exactly which downstream signaling pathways mediate these processes remain to be elucidated in forthcoming studies.

## Defective transduction of fluid flow as a basis for pathogenesis of HHT

The primary cellular defects underlying AVM genesis are unknown. Animal studies suggest that AVMs form during a period of dynamic vascular remodeling, such as, during developmental angiogenesis or when quiescent ECs are activated in response to wound-induced vascular remodeling or tissue regeneration (Lasjaunias, 1997; Seki et al., 2003; Park et al., 2009; Shovlin, 2010; Corti et al., 2011; Rochon et al., 2016; Sugden et al., 2017). The formative stages of vascular development, during which nascent vascular tubes are perfused for the first time, appears to be a critical setting for the genesis of AVMs and telangiectasias (Corti et al., 2011; Baeyens et al., 2016; Franco and Gerhardt, 2016; Rochon et al., 2016; Jin et al., 2017; Sugden et al., 2017; Ola et al., 2018).

The fluid flow that ensues the first cardiac contractions in the fetus provide the hemodynamic force and the growth/humoral factors that contribute to artery-vein specification, establishment of a hierarchically ordered and lumenized vasculature (reviewed by Red-Horse and Siekmann, 2019), and vessel stabilization, through facilitating perivascular cell coverage (Van Gieson et al., 2003; Ando et al., 2016; Chen et al., 2017; Padget et al., 2019). Therefore, the capacity of nascent ECs to accurately detect and transduce the magnitude and direction of these initial flow patterns is crucial for the establishment of stable arteriovenous connections. Emerging evidence on HHT pathobiology in animal models and human-derived ECs have converged on a common theme, involving defective flow transduction as a basis for the development of AVMs: Loss of components of TGF-β signaling impairs arteriovenous development, in part, due to defective fluid shear stress transduction or impaired mechanotransduction (Corti et al., 2011; Baeyens et al., 2016; Franco and Gerhardt, 2016; Rochon et al., 2016; Jin et al., 2017; Sugden et al., 2017; Ola et al., 2018).

For instance, endothelial-specific deletion of SMAD4 in postnatal mice results in abnormal EC proliferation and AVMs that lack mural cell coverage, predominantly in regions where the vasculature experiences high blood flow (Ola et al., 2018). This suggests that ECs lacking SMAD4, the downstream effector of ALK1, display pathological responses to fluid flow. Similarly, in an embryonic zebrafish model of Alk1 loss-of-function, Corti et al. (2011) have shown that induction of cerebral blood flow in *alk1*−/− embryos results in abnormal increases in EC numbers, followed by progressive emergence of AVM-like shunting in the hindbrain vasculature (Corti et al., 2011). This observation indicates that an interaction between genetic propensity and hemodynamic stimulus contributes to pathogenesis of these vascular malformations, particularly, at a specific developmental period (Corti et al., 2011). In fact, cessation of blood flow in *alk1−/−* embryos was sufficient to prevent abnormal shunting and AVM formation (Corti et al., 2011). In a follow-up study by the same group, Rochon et al. (2016) revealed that transient knockdown of *alk1* in embryonic zebrafish impairs flow-induced migration of cranial ECs towards the heart, attesting to a requirement for *Alk1* for mediating endothelial responses to flow (Rochon et al., 2016). Consistently, postnatal deletion of *Alk1* in murine ECs is associated with AVM genesis in vessels exposed to higher blood flow velocities than in more primitive vascular plexus, which tend to sustain more sluggish blood flow (Baeyens et al., 2016; Franco and Gerhardt, 2016). This suggests that ALK1 is required for flow transduction and mediating endothelial flow responses in vertebrates.

More recently, Sugden et al. (2017) have shown, using zebrafish ENG orthologous mutants (*eng*−/−), that *eng−/−* ECs, unlike their wild-type siblings, assume a disorganized morphology following flow induction and that cessation of flow was sufficient to rescue these abnormal endothelial morphologies in *eng−/−* ECs (Sugden et al., 2017). Similarly, work done by Jin et al. (2017) in human dermal microvascular ECs (HDMECs) and lung ECs derived from *Eng* loss-of-function mutant mice, revealed that *Eng* expression is required for normal directional migration of ECs subjected to laminar fluid flow (Jin et al., 2017). Given that mouse embryonic ECs with *Eng* loss-of-function have been shown to display impaired trans-endothelial barrier function (Jerkic and Letarte, 2015), it is expected that an increase in flow velocity and/or defective flow transduction would exacerbate the hyper-permeability observed in *ENG* mutant ECs.

Collectively, studies in diverse model systems underscore a common theme, namely, the inability of ECs to withstand, transduce and respond to hemodynamic stimuli, particularly during early vascular development, will result in aberrant endothelial responses that give rise to malformed arteriovenous connections. Similarly, an abnormal surge in shear stress (due, for example, to factors such as a surge in blood pressure, vascular injury or changes in blood viscosity) can trigger maladaptive endothelial responses that precipitate conditions for AVM genesis in specific vessel beds (Tu et al., 2014; Thomas et al., 2016). But how do ECs sense and communicate changes in fluid force magnitude and direction to potentiate ALK1/SMAD4 signaling and regulate vascular stabilization at the same time?

In the following section, we hypothesize, by integrating our recent findings with emerging evidence from the literature, that impaired flow transduction, as a consequence of premature loss or dysfunction of endothelial primary cilium, a microtubule-based flow-sensitive organelle, constitutes one of the earliest steps in the pathogenesis of some forms of AVMs.

## Hypothesis: Embryonic loss of endothelial cilia will lead to AVMs through curtailment of TGF-β signaling

The expression of ALK1 is positively correlated with blood flow (Seki et al., 2003; Corti et al., 2011; Laux et al., 2013; Baeyens et al., 2016; Rochon et al., 2016). However, it remains unknown if there are upstream endothelial flow detectors that relay information about flow intensity/patterns and concomitantly potentiate the canonical BMP9→ALK1/ENG→SMAD4 mediated vascular morphogenesis. ECs, by virtue of their intimate association with blood flow (*tunica intima*), have multiple flow-sensitive components and membrane invaginations that are crucial for adaptive responses, some of which include the caveolae, membrane-associated mechano-sensitive ion channels, the glycocalyx, components of extracellular matrix, cell-cell junction proteins, G-protein coupled receptors and the primary cilium (Reviewed by Chien, 2007; Givens and Tzima, 2016; Shin et al., 2019; Fang et al., 2019).

Of these endothelial structures, the primary cilium, a microtubule-based cellular protrusion that can reach a variable length of 1–11 μm, depending on culture conditions (Van Der Heiden et al., 2008; Lim et al., 2015; Dummer et al., 2016), is the most distally extending EC organelle and is, likely, the most intimately associated with blood flow through the lumen. Primary cilia have been observed in ECs derived from post-mortem human embryonic aortas and adult human aortic arches (atherosclerotic lesions, in particular) (Bystrevskaya et al., 1988). Interestingly, cilia in the embryonic aorta were found to be either immersed in the cytoplasm or facing the abluminal side (Bystrevskaya et al., 1988). However, the functional relevance of these hair-like organelles and their distribution in ECs had been a mystery at the time: “It is being stressed that whereas the significance of these unusual organelles remains uncertain, their widespread occurrence may indicate that their role is more important than was believed previously, and they should cease being a curiosity only” (Haust, 1987). Primary cilia can be connected to cytoskeletal elements that run along the length of the cell (Hierck et al., 2008; Jones et al., 2012), and are thought to be organizing platforms, harboring various mechano-sensitive ion channels, membrane proteins and receptors (Nauli et al., 2008; Lee et al., 2015).

In culture, ECs derived from a mouse model of ciliopathy (Tg737^orpk/orpk^) exhibit defects in directional migration and display enhanced trans-endothelial permeability *in-vitro* (Jones et al., 2012). *In-vivo*, we and others have shown that primary cilia are enriched during the emergence of the nascent vasculature in both the head and trunk regions of embryonic zebrafish (Goetz et al., 2014; Eisa-Beygi et al., 2018). Endothelial cilia numbers decrease in post-embryonic zebrafish, concomitant with increases in cardiac output and the accompanying surge in flow velocity and shear stress levels (Goetz et al., 2014; Eisa-Beygi et al., 2018). Similarly, whereas cilia are enriched in embryonic human aorta, adult human aortic arches are almost completely devoid of primary cilia (Bystrevskaya et al., 1988). In post-embryonic zebrafish and mice, endothelial cilia are scarce and appear to be confined to distinct regions of the vasculature that experience more disturbed flow patterns, such as inner regions of curved, bifurcating or branching vessel beds (Van Der Heiden et al., 2008; Dinsmore and Reiter, 2016; Eisa-Beygi et al., 2018). This suggests that endothelial cilia are specialized to detect and transduce low magnitudes of fluid forces, such as those exerted during the onset of circulation in early development (Goetz et al., 2014) or in vessels experiencing low or disturbed flow regimes due to their non-linear topographies or contours. Shear stress levels below 10–15 dyn/cm^2^ appear to be conducive to ciliation of ECs and their optimum mechanotransduction function (Iomini et al., 2004). This observation agrees with findings in cultured EC monolayers, in which exposure to high shear stress leads to shedding or disassembly of primary cilia (Iomini et al., 2004). In contrast, ECs raised under static or low shear stress conditions retain their cilia (Vion et al., 2018). Hence, *in-vitro* and *in-vivo* evidence suggest that endothelial cilia are specialized organelles for detection of low flow profiles.

During zebrafish development, endothelial cilia are widely distributed in newly emerging head vasculature, preceding lumen formation and flow inception (Eisa-Beygi et al., 2018). Endothelial cilia are also observed during angiogenesis of hindbrain arteries, as arterial-fated endothelial sprouts emerge from the bilaterally located venous channels, migrate dorsa-medially, anastomose with the centrally located basilar artery to establish perfused arteriovenous connections in the hindbrain (Eisa-Beygi et al., 2018). Consistently, genetic and pharmacological loss of components of cilia biogenesis in embryonic zebrafish elicit intracerebral hemorrhages (Lamont et al., 2010; Ben et al., 2011; Arnold et al., 2015; Kallakuri et al., 2015; Eisa-Beygi et al., 2018), miss-patterned arteriovenous connections in the hindbrain (Ben et al., 2011) and reduced number of contacts between ECs and the surrounding perivascular cells (Lamont et al., 2010). These observations agree with recent findings, which suggest that endothelial cilia facilitate recruitment of perivascular cells to arterial-fated blood vessels in embryonic zebrafish, in a flow-mediated mechanism (Chen et al., 2017).

In contrast to observations made in embryonic zebrafish, in post-embryonic zebrafish, endothelial-specific deletion of cilia does not appear to affect vascular patterning in juvenile or adult zebrafish (Elworthy et al., 2019). Collectively, these findings allude to the possibility that endothelial cilia are required during early embryonic development to transduce the initially low flow patterns that ensue the first cardiac contractions in the embryo, thereby mediating the transition from a naïve vascular plexus into a lumenized, perfused, ordered, and stable arteriovenous network.

Studies in murine models support a potential function for endothelial cilia in vascular development. Liu et al. (2018) have shown, in an angiotensin II-elastase model of intracranial aneurysm, that mice with defects in cilia biogenesis were more prone to develop intracranial aneurysms and with a higher incidence of rupture, than their wild-type siblings subjected to the same treatment (Liu et al., 2018). Dinsmore and Reiter (2016) have shown that whereas endothelial loss of a key component of cilia biogenesis did not induce any vascular abnormalities, these mice were more likely to develop atherosclerosis, suggesting that the presence of cilia negatively correlates with atherosclerotic lesion development (Dinsmore and Reiter, 2016). More recently, Vion et al. (2018) have revealed that loss of endothelial cilia during postnatal development is associated with impaired sprouting, vessel regression and lumen collapse in mice retinal vasculature (Vion et al., 2018).

**FIGURE 1:**
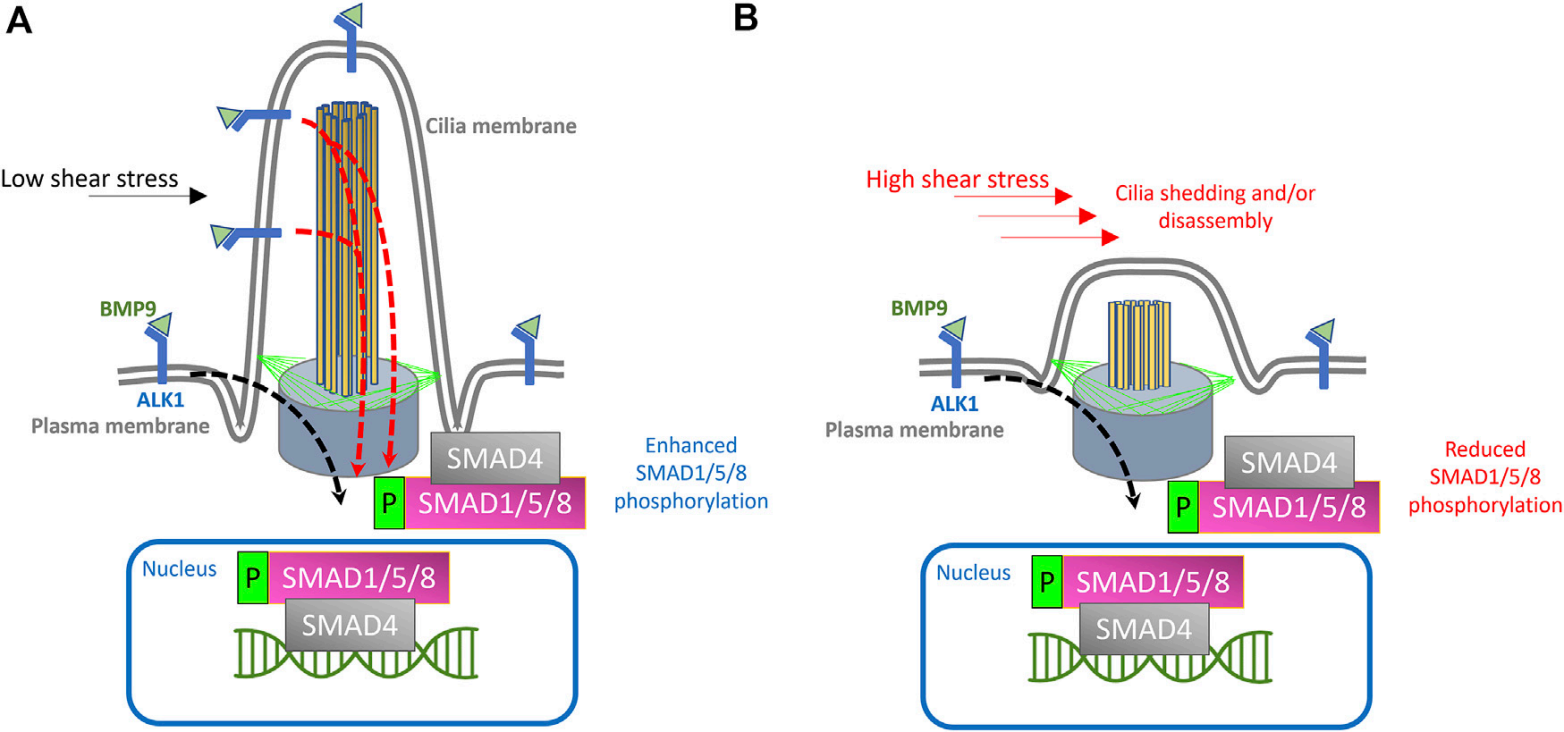
Proposed model for endothelial cilia mediated BMP/ALK1/SMAD signaling. **(A)** Schematic depiction of endothelial primary cilium, showing microtubule-based axoneme (yellow rods), basal body (purple cone), transition fibers (green lines) and cilia membrane (labeled). In the proposed model, ALK1 is enriched both at the plasma membrane and at the ciliary membrane. BMP9 is available under low shear stress conditions through circulation and binds to ALK1 in the EC membrane and at the ciliary membrane. Under low shear stress, the binding of BMP9 to ALK1 will result in the phosphorylation of SMAD1/5/8 and translocation of SMAD4 + phosphorylated SMAD1/5/8 complex to the nucleus, where this complex will act as a transcription factor to drive the expression of genes required for pericyte recruitment and vessel stabilization, giving rise to stable arteriovenous connections. **(B)** Exposure to high shear stress would result in cilia disassembly or dysfunction, thereby curtailing cilia mediated ALK1 signaling and downstream processes.

Taken together, animal studies suggest that endothelial cilia may have a functional role in mediating several aspects of vascular development in teleosts and mammals, with potential involvement in sprouting angiogenesis, lumenization, recruitment of perivascular cells, and vessel stabilization. Published observations suggest that these morphogenic events are mediated, in part, *via* cilia-mediated potentiation of TGF-β signaling. Using human-derived vein endothelial cells (HUVECs), Vion et al. (2018) have recently shown that several components of the canonical TGF-β signaling, namely, ALK1, p-SMAD1/5/8, and SMAD4, are co-localized to the primary cilium (Vion et al., 2018). They further showed that loss of intraflagellar transport protein 88 (IFT88), crucial for cilia biogenesis, inhibits ALK1 signaling (Vion et al., 2018). This agrees with previous work done on human fibroblasts, where SMAD4 was shown to be restricted to the ciliary base (Clement et al., 2013). Similarly, in cardiomyocytes differentiated from mouse embryonic stem cells, SMAD4 is localized to primary ciliary pocket (Koefoed et al., 2014). In human mesenchymal stem cells (hMSCs), SMAD4 is shown to be concentrated along the length of the primary cilium (Labour et al., 2016). Other components of TGF-β signaling have also been detected in and around primary cilium in human-derived fibroblasts, mesenchymal stem cells, and embryonic stem cells (hESCs) (Clement et al., 2013; Labour et al., 2016; Vestergaard et al., 2016; Pala et al., 2017). More recently, Villalobos et al. (2019) have shown that loss of cilia impairs TGF-β signaling in cardiac fibroblasts (Villalobos et al., 2019). These studies evoke the possibility that cilia serve as conjunction points or scaffolds for facilitating the canonical TGF-β signaling in ECs that are subjected to low flow velocities, with the initial signaling cascade taking place at both the endothelial membrane and ciliary compartment (Figure 1A). On balance, the model we propose is as follows:

On balance, the model we propose is as follows:

1) Endothelial primary cilia membrane harbors components of ALK1 signaling.
2) During angiogenesis, tip cells exhibit primary cilia as they migrate and fuse with neighboring sprouts.
3) Following tip cell anastomosis, junctional remodeling and lumenization, endothelial primary cilia serve as signaling hubs to increase sensitivity of ECs to BMP9/10 gradients under low shear stress conditions
4) Loss or dysfunction of primary cilia (e.g., due to abnormal surge in shear stress or defects in cilia assembly) during this stage would result in a concordant reduction in BMP9/10 sensitivity and inhibit cellular processes mediated by the canonical TGF-β signaling in ECs, resulting in misguided angiogenesis of the capillary network.

## Conclusion and future directions

**FIGURE 2:**
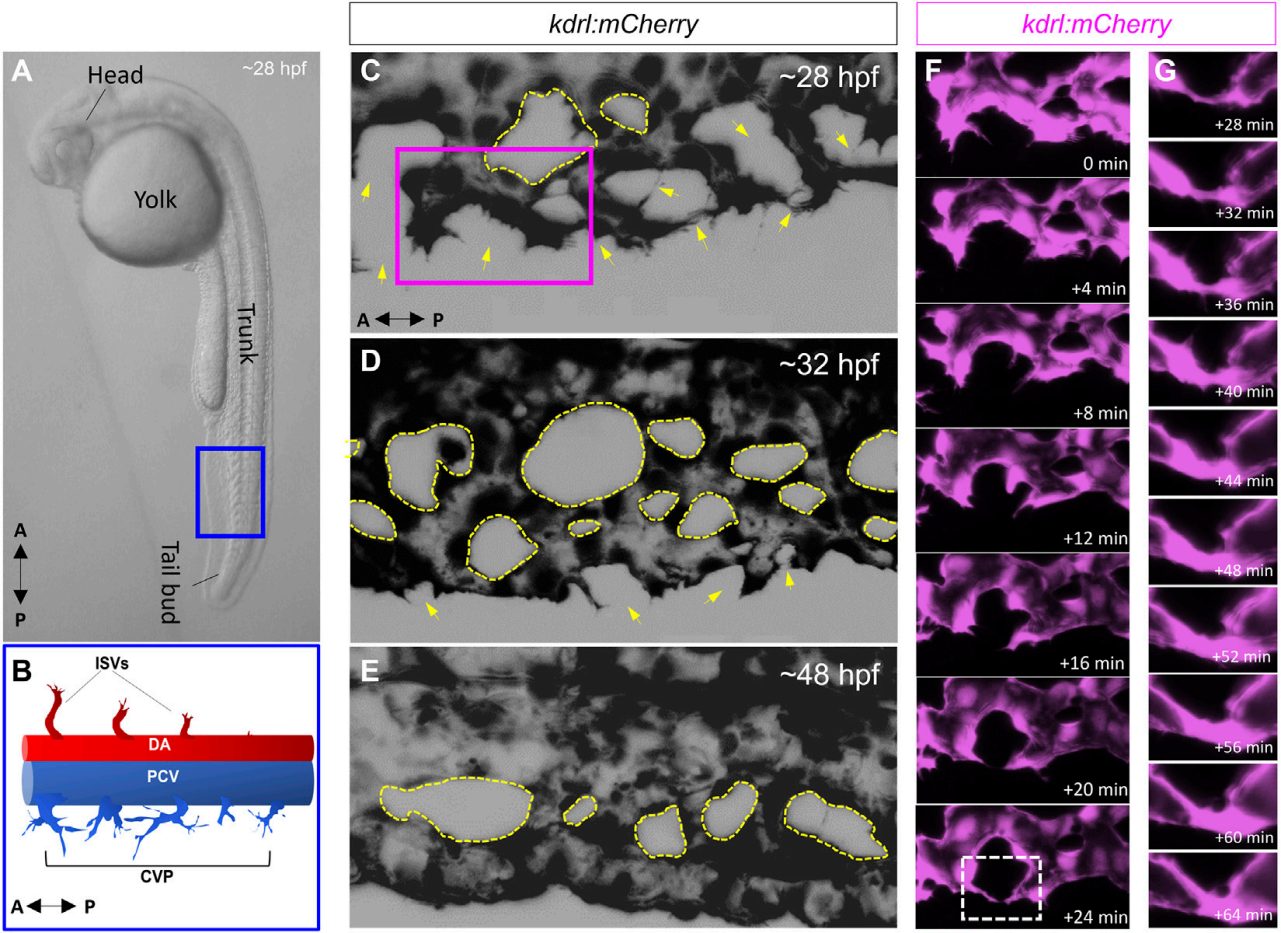
The zebrafish caudal vein plexus (CVP) is formed *via* BMP mediated angiogenesis. **(A)** Representative photomicrograph of a 28-h post fertilization (hpf) zebrafish embryo. Lateral view is shown. **(B)** Schematic depiction of the two conserved axial vessels in the blue boxed region in A, depicting the dorsal artery (DA) and posterior caudal vein (PCV). The arterial-fated intersegmental vessels (ISVs) emanate from the dorsal surfaces of DA and migrate dorsally in response to Vegf gradients. The PCV gives rise to venous sprouts that migrate ventrally in response to Bmp gradients to form the caudal vein plexus (CVP). **(C–E)** Representative maximum projections of the CVP during successive stages of sprouting, anastomosis and remodeling. The yellow arrows in **(C)** and **(D)** point to putative sites of tip cell fusion or anastomosis **(F)** Higher magnification of the purple boxed region in C, showing fusion of ipsilateral tip cells **(G)** Digital zoom of the white boxed region in F, showing progressive lumenization of site of anastomosis.

**FIGURE 3:**
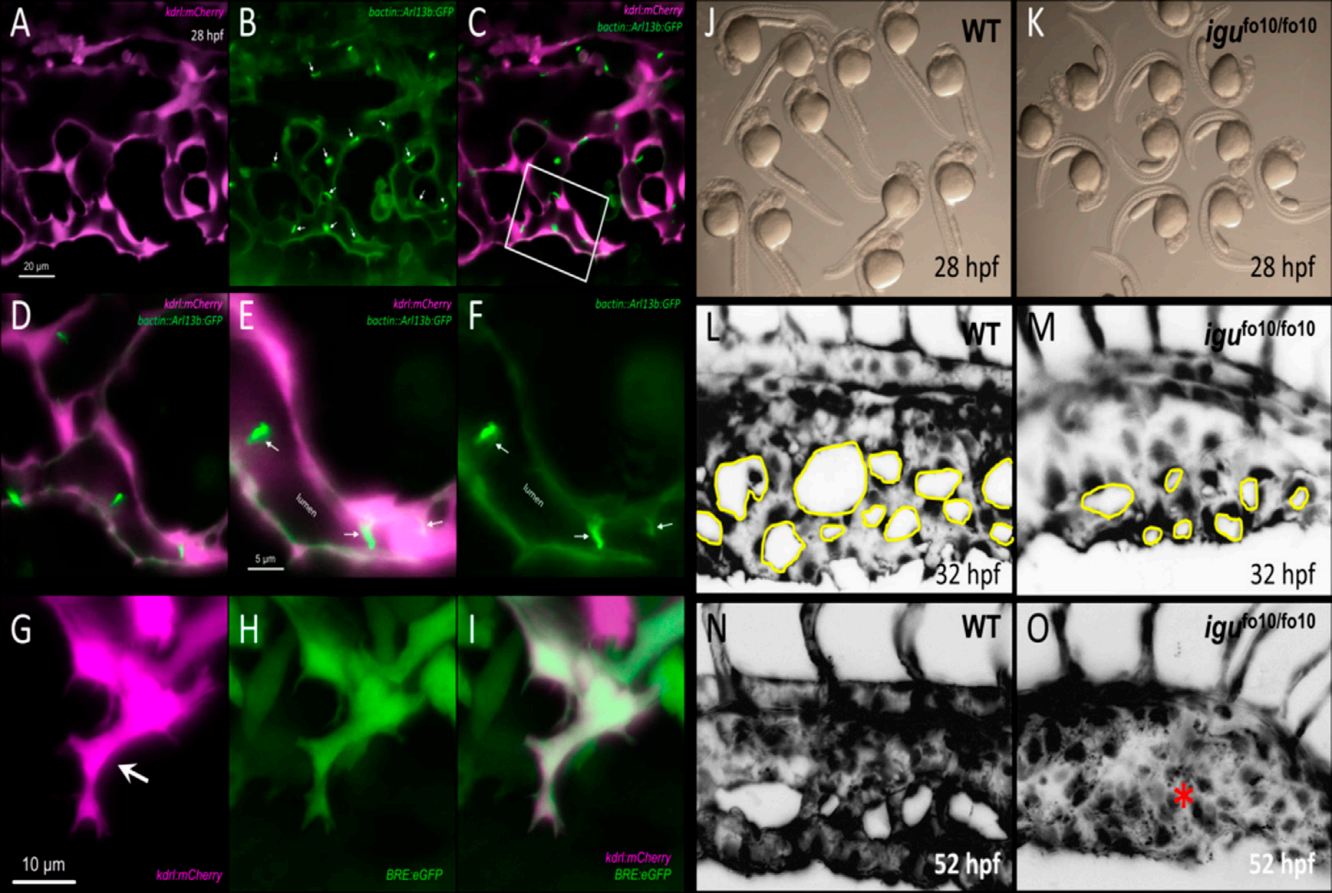
Involvement of primary cilia in the emergence of the caudal vein plexus (CVP). **(A–C)** Representative single stack confocal images of the CVP region in the *Tg*(*kdrl:mCherry-CAAX)* ^*y171*^ *;*(*bactin::Arl13b:GFP)* double transgenic embryo at approximately 28 h post fertilization (hpf). ECs express mCherry and primary cilia are labeled by GFP expression. **(D–F)** Higher magnification of the white boxed region in C. White arrows point to primary cilia. **(G–I)** Representative maximum projection confocal images of a venous sprout in the CVP region at approximately 28 hpf in the *Tg*(*kdrl:mCherry-CAAX)* ^*y171*^; (*BRE:eGFP)* double transgenic embryom showing endothelial cells (mCherry) and BMP-responsive (BRE+) cells (eGFP) at approximately 28 hpf. White arrow points to a tip cell **(J)** Representative photomicrograph of wild-type embryos at approximately 28 hpf. **(K)** Representative photomicrographs of *igu* ^fo10/fo10^ embryos which have a loss-of-function mutation in the ciliary basal body protein, Dzip1. Lateral images are shown. **(L)** Representative maximum projection image of the developing caudal vein plexus (CVP) in a wild-type embryo at 32 hpf **(M)** Representative maximum projection image of the developing CVP region in an *igu* ^fo10/fo10^ embryo at 32 hpf. **(N)** Representative maximum projection image of the CVP in a wild-type embryo at 52 hpf **(O)** Representative maxmum projection image of the CVP in *igu* ^fo10/fo10^ embryo, showing malformed CVP, evidenced by lack of CVP loops and an overall dilated morphology. This is depicted with a red asterisk. All images are lateral.

We predict that premature loss of endothelial cilia during development, particularly at a time when immature ECs coalesce to form a newly perfused vascular tube, would curtail transduction of initial flow patterns to ECs. This would result in attenuation of TGF-β signaling and curtailment of downstream EC autonomous processes needed for vascular stabilization (Figure 1). Several questions remain to be addressed in order to further expound on the contribution of endothelial cilia to ALK1/SMAD4 mediated vascular development:

1- Improved imaging modalities and post-processing of images to rigorously quantify and colocalize components of ALK1/ENG→SMAD4 signaling are needed to convincingly demonstrate their physical association with the endothelial primary cilium. Specifically, better imaging modalities and image analysis, as well as techniques such as rapid protein proximity labeling can be employed to accurately co-localize primary cilia with components of ALK1/SMAD4 signaling (Pino and Schilling, 2021; Tameling et al., 2021).
2- If endothelial cilia are indeed conducive to BMP9/ALK1/SMAD4, we would predict that ECs lacking primary cilia (due, for example, to increased flow velocity or defective cilia assembly) would have significantly attenuated ALK1 signaling, as evidenced by reduced phosphorylation rates of SMAD1/5/8. Interestingly, under high shear stress conditions that would otherwise result in deciliation, ECs are still able to display synergistic interaction between shear stress levels and translocation of p-SMAD1/5/8 to the nucleus following exogenous BMP9 supplementation (Vion et al., 2018). However, in ECs lacking IFT88, a crucial cilia protein, this translocation is reduced under similar shear stress levels (Vion et al., 2018). To further test this prediction, immunohistochemistry and/or Western blot analysis can be performed to specifically determine the expression and distribution of the phosphorylated levels of SMAD1/5/8 (Kautz et al., 2008). Additionally, changes in the levels of SMAD-mediated BMP signaling can be evaluated *in-vivo*, using transgenic zebrafish reporter lines that our lab had previously generated (Collery and Link, 2011). In particular, in the *Tg(BRE:dsGFP)* line, the BMP response element (BRE) derived from mouse Id1 promoter, which includes multiple Smad-binding elements, drives the expression of destabilized GFP, thus enabling *in-vivo* assessment of the canonical BMP/Smad1/5/8-mediated signaling (Collery and Link, 2011). The destabilized GFP, due to its rapid turnover, provides a more sensitive readout of dynamic BMP responses in cells (Li et al., 1998; Collery and Link, 2011).
3- Similarly, we predict that premature loss or dysfunction of endothelial cilia will impair translocation of p-SMAD1/5/8 and SMAD4 to the nucleus, where they would otherwise induce transcription of genes required for vascular stabilization and EC quiescence (Figure 1). In ECs lacking primary cilia, we predict reduced translocation of the p-SMAD1/5/8 and SMAD4 complex to the nucleus and reduced transcription of their target genes (Taylor and Khachigian, 2000; Li et al., 2011).
4- There have been a limited number of isolated case reports of ciliopathy patients presenting with AVMs and arteriovenous fistulas in the brain, the cervix and the lungs (Banerjee and Rana, 1976; Proesmans et al., 1982; Tsuruda et al., 1996; Nishida et al., 2002; Bit et al., 2012; Grieb et al., 2014; Aregullin et al., 2019). Nonetheless, there does not appear to be a consistently established link between ciliopathies and AVMs or other vascular malformations in animal models or in the clinic. The zebrafish offers an excellent *in-vivo* paradigm to study BMP-mediated angiogenesis. By 24 h post fertilization (hpf), zebrafish embryos exhibit the two conserved axial vessels along the medial side of the somites, namely, the dorsal aorta (DA) and the ventrally apposed posterior cardinal vein (PCV) (Figures 2A,B). The angiogenic sprouts emerging from the dorsal surfaces of DA migrate ventrally along somite boundaries in response to vascular endothelial growth factor (Vegf) (Kim et al., 2014). In contrast, the PCV-derived venous tip cells respond specifically to Bmp gradients and migrate ventrally (Kim et al., 2014). Starting at 25–26 hpf, venous ECs branch out from the ventral ends of the caudal portions of PCV, migrating ventrally as they extend multiple F-actin based filopodial protrusions (Wakayama et al., 2015). At the ventromedial boundary, ipsilateral tip cells fuse with adjacent sprouts through a process called anastomosis, followed by lumenization, giving rise to a provisional network known as the caudal vein plexus (CVP) (Figures 2C–G). By ∼32 hpf, a network of venous tubes, with multiple inter-capillary spaces, also known as CVP loops, is formed (Figure 2D). The formation of CVP depends primarily on fusion and reorganization of tip cells at the angiogenic front, followed by establishment of patent circuits and remodeling through pruning (Figure 2E). Despite insights into how venous tip cells initiate sprouting and migration in response to Bmp gradients, the mechanisms governing their timely and precise fusion with neighboring sprouts remain unclear. Through high-resolution imaging of the *Tg*(*kdrl:mCherry-CAAX)* ^*y171*^ *;* (*bactin::Arl13b:GFP)* line, we have shown that the ECs making up these sprouts manifest primary cilia as they migrate ventrally (Figures 3A–F). Furthermore, these venous sprouts show are responsive to BMP, as revealed using a BMP reporter line (Figures 3G–I). However, the precise function of these cilia in sprouting tip cells and whether they are instrumental in enhancing the sensitivity of ECs to BMP gradients remains to be addressed. Next, given the preponderence of cilia at the angiogenic front of CVP, we evaluated the formation of CVP in a previously identified zebrafish model of ciliopathy, namely, the *iguana* (*igu* ^fo10/fo10^) mutants (Arnold et al., 2015). The *igu* ^fo10/fo10^ mutants have a loss-of-function mutation in the ciliary basal body protein, Dzip1. In addition to a curved tail phenotype and brain hemorrhages reported previously (Arnold et al., 2015), we have observed that by 30 hpf, almost all homozygote mutants displayed defective angiogenesis of CVP, evidenced by impaired sprouting and anastomosis events, followed by vessel collapse, a dilated phenotype and blood pooling by 52 hpf (Figures 3J–O). Consistent with our observations in *iguana* mutants, Goetz et al. (2014) observed similar defects on the morphogenesis of CVP following regular and conditional knockdown of polycystin-2 (*pkd2*) or *ift88*, due primarily to “decreased number and activity of venous sprouts” (Goetz et al., 2014). More granular studies are warranted to specifically test a functional role for endothelial primary cilia on aspects of CVP formation, with a particular focus on parameters such as tip cell induction, sprout migration pattern, tip cell filopodial actin dynamics through the use of reporter lines such as *Tg(fli1:lifeact-mCherry)* (Wakayama et al., 2015), endothelial cell numbers *via* endothelial nuclear-specific line such as *Tg*(*kdrl:nls-mcherry)* ^*y137*^(Fujita et al., 2011), number of anastomosing vessels, capillary diameter, *in-vivo* assessment of shunting formation, and vascular permeability using fluorescent dyes or erythrocyte-specific transgenic lines such as *Tg(gata1:dsRed)* (Traver et al., 2003).

Although these observations do not address a specific role for endothelial cilia in mediating arteriovenous development, they do point to a potential involvement in developmental angiogenesis, which needs further characterization through endothelial-specific inhibition of cilia biogenesis. Intriguingly, although both global transient knockdown and heterozygote loss of *bmp9* were shown to impair CVP formation in embryonic zebrafish, the observed phenotypes are not as severe as those observed in *iguana* mutants (Wooderchak-Donahue et al., 2013; Li et al., 2020). Further adding to the phenotypic inconsistency, a recent study did not report any discernable vascular defects in *bmp9* mutants (Capasso et al., 2020). However, both Bmp9 and Bmp10, ligands for Alk1, are secreted into the blood stream, and cessation of blood flow in embryonic zebrafish results in complete inhibition of CVP angiogenesis (Goetz et al., 2014). Therefore, future studies are warranted to ascertain how flow contributes to the spatio-temporal distribution of Bmp9/10 in the developing vasculature and whether presence of cilia enhances endothelial responsiveness to these ligands. Also, previous phenotypic characterization of Alk1 loss-of-function in zebrafish did not report any defects in CVP formation or cerebral-vascular permeability, although the embryos displayed progressive shunting and dilation in cerebral vessels (Rochon et al., 2016).

However, one of the most common vascular manifestations of ciliopathies are hemorrhage-prone intracranial aneurysms, as observed in patients with autosomal dominant polycystic kidney disease (ADPKD), an inherited ciliopathy condition (Chapman et al., 1992; Pirson et al., 2002; Rozenfeld et al., 2014). Whether the brain aneurysms commonly associated with ADPKD arise, at least in part, due to defects in arteriovenous development, has, to our knowledge, not been addressed. This possibility, coupled with the fact that some AVMs appear to arise sporadically, with no known germline or somatic mutations, suggest that premature loss or dysfunction of endothelial cilia during vascular morphogenesis and remodeling may precipitate the primary conditions for AVM genesis. Therefore, we speculate that AVMs could be part of the expanding spectra of vascular defects associated with ciliopathies.

## Data Availability

The raw data supporting the conclusion of this article will be made available by the authors, without undue reservation.

## Abbreviations

ADPK, Autosomal dominant polycystic kidney disease; AVM, Arteriovenous malformation; ALK1, Activin A receptor-like kinase 1; BMP9, Bone morphogenic protein-9; BMP10, Bone morphogenic protein-10; CNS, Central nervous system; ENG, Endoglin; GFP, Green fluorescent protein; HDMECs, Human dermal microvascular endothelial cells; hESCs, Human embryonic stem cells; HHT, Hereditary hemorrhagic telangiectasia; hMSCs, Human mesenchymal stem cells; HUVEC, Human umbilical vein endothelial cells; SMAD4, Mothers against decapentaplegic homolog 4; TGF-β, Transforming growth factor-beta; p-SMAD1/5/8, Phosphorylated SMAD1/5/8.
